# Spatial distribution and determinant factors of anemia among adults aged 15–59 in Ethiopia; using mixed-effects ordinal logistic regression model

**DOI:** 10.1186/s40795-021-00424-4

**Published:** 2021-05-20

**Authors:** Biruk Shalmeno Tusa, Sewnet Adem Kebede, Adisu Birhanu Weldesenbet

**Affiliations:** 1 Department of Epidemiology and Biostatistics, College of Health and Medical Sciences, Haramaya University, Haramaya, Ethiopia; 2 Department of Epidemiology and Biostatistics, Institute of Public Health, College of Medicine and Health Sciences, University of Gondar, Gondar, Ethiopia

**Keywords:** Anemia, Adults, Spatial analysis, Mixed-effects ordinal logistic regression, Ethiopia

## Abstract

**Background:**

Anemia is a global public health problem, particularly in developing countries. Assessing the geographic distributions and determinant factors is a key and crucial step in designing targeted prevention and intervention programmes to address anemia. Thus, the current study is aimed to assess the spatial distribution and determinant factors of anemia in Ethiopia among adults aged 15–59.

**Methods:**

A secondary data analysis was done based on 2016 Ethiopian Demographic and Health Surveys (EDHS). Total weighted samples of 29,140 adults were included. Data processing and analysis were performed using STATA 14; ArcGIS 10.1 and SaTScan 9.6 software. Spatial autocorrelation was checked using Global Moran’s index (Moran’s I). Hotspot analysis was made using Gettis-OrdGi*statistics. Additionally, spatial scan statistics were applied to identify significant primary and secondary cluster of anemia. Mixed effect ordinal logistics were fitted to determine factors associated with the level of anemia.

**Result:**

The spatial distribution of anemia in Ethiopia among adults age 15–59 was found to be clustered (Global Moran’s I = 0.81, *p* value <  0.0001). In the multivariable mixed-effectordinal regression analysis; Females [AOR = 1.53; 95% CI: 1.42, 1.66], Never married [AOR = 0.86; 95% CI: 0.77, 0.96], highly educated [AOR = 0.71; 95% CI: 0.60, 0.84], rural residents [AOR = 1.53; 95% CI: 1.23, 1.81], rich wealth status [AOR = 0.77; 95% CI: 0.69, 0.86] and underweight [AOR = 1.15; 1.06, 1.24] were significant predictors of anemia among adults.

**Conclusions:**

A significant clustering of anemia among adults aged 15–59 were found in Ethiopia and the significant hotspot areas with high cluster anemia were identified in Somalia, Afar, Gambella, Dire Dewa and Harari regions. Besides, sex, marital status, educational level, place of residence, region, wealth index and BMI were significant predictors of anemia. Therefore, effective public health intervention and nutritional education should be designed for the identified hotspot areas and risk groups in order to decrease the incidence of anemia.

## Background

Anemia is a condition characterized by a low blood hemoglobin concentration (below 130 g/L for men, 120 g/L for non-pregnant women and below 110 g/L in pregnant women). In such condition the oxygen-carrying capacity of Red blood cells (RBCs) is insufficient to meet physiological needs. It is a global public health problem, particularly in low- and middle-income countries [1, 2].

Over a quarter of the world’s population is affected by anemia. Anemia can affect all age groups and has the highest prevalence in preschool-age children (47.4%), the lowest prevalence in men (12.7%) and the greatest number of individuals affected among non-pregnant women (468.4 million) [3, 4]. In Africa, prevalence rates of 16.8 to 33.8% among adults were reported in Uganda [5] whereas another study in South Africa [6] indicated anemia prevalence of 12.5% in males and 13.2% in females. In Ethiopia, the prevalence of 13% was reported among adults of both sexes [7].

Micronutrient deficiencies, parasitic infection, infectious diseases like TB, HIV, and maternal blood loss are common causes of anemia in developing countries. The occurrence of the risk factors of anemia concomitantly makes the efforts to single out a definitive cause challenging, especially in settings lacking resources [8–10]. Drug toxicity, deficiencies of iron, certain vitamins, copper, and protein are additional contributing factors for anemia and Iron deficiency is the most common cause of anemia responsible for 75% of anemia cases [11].

Anemia has large negative consequences on the health and economic wellbeing of nations and communities [12]. In Ethiopia, despite the efforts made to reduce anemia at a national level, it is remained still an important public health problem for it is affected by geographical variation [13]. Assessing the geographic distributions of anemia and determinant factors are key and crucial step in designing targeted prevention and intervention programmes that are meant to address anemia. Moreover, mapping the spatial distribution of anemia by regions can help focus resources for prevention and treatment in the hot spot areas. Therefore, this study is aimed to assess the spatial distribution and determinant factors of anemia in Ethiopia among adults aged 15–59 using mixed-effect ordinal logistic regression.

## Methods

### Study setting and data source

Ethiopia is one of the East African countries which contained nine National, Regional states: namely Tigray, Afar, Amhara, Oromia, Somali, Benishangul-Gumuz, Southern Nations, Nationalities and People Region (SNNPR), Gambella and Harari, and two Administrative states (Addis Ababa City administration and Dire Dawa city council). This study was a secondary data analysis based on 2016 Ethiopian Demographic and Health Surveys (EDHS). The 2016 EDHS provides reliable estimates at the national level, for urban and rural areas, and for each of the 9 regions and 2 administrative cities [14].

The EDHS 2016applied a stratified two-stage cluster sampling technique (By considering the 2007 Population and Housing Census (PHC) as a sampling frame). Stratification was realized by separating each region into urban and rural areas. Accordingly, a total of 21 sampling strata had been formed. In the first stage, 645 Enumeration Areas (EAs) (202 in urban areas and 443 in rural areas) were selected with probability proportional to the enumeration area size and with independent selection in each sampling stratum. At the second stage, since the time has passed since the PHC, a complete household listing operation was carried out in all selected EAs before the start of fieldwork and on average 28 households were selected per enumeration area.

In the EDHS 2016, Blood specimens for anemia testing were collected from women and men aged 15–59 that voluntarily consented to be tested. Blood samples were drawn from a drop of blood taken from a finger prick and collected in a micro cuvette. Hemoglobin analysis was carried out on site using a battery-operated portable HemoCue analyzer. The full sampling procedure and the Anemia testing data are accessible in the full 2016 EDHS report [14]. The total weighted samples of 29,140 adults were included in this study.

### Study variables

The outcome variable for this study was hemoglobin level in the blood, a key indicator for Anemia. Hemoglobin levels (after adjusting for cigarette smoking and altitude in enumeration areas that are above 1000 m) in which women and men are considered not anaemic, mild, moderate and severe anaemic were presented in Table 1.

**Table 1:** Description of outcome variable used in the Analysis

Anemia levels	Respondents (Hemoglobin level in g/dl)		
	Pregnant women	Non-pregnant women	Men
Not anaemic	≥ 11.0	≥ 12.0	≥ 13.0
Mild	10.0–10.9	10.0–11.9	10.0–12.9
Moderate	7.0–9.9	7.0–9.9	7.0–9.9
Sever	< 7.0	< 7.0	< 7.0

Sex, age, marital status, educational level, place of residence, region, wealth index, source of drinking water, type of toilet facility, and Body Mass Index (BMI) were included as independent variables in this study.

### Operational definition

**Adult:** Individuals aged greater than or equal to 15 were considered as adult in our study.

**Wealth index:** it is the percent distribution of the dejure population by wealth quintiles and the Gini coefficient. It was classified as Poorest, Poorer, Middle, and Richer & Richest.

**Uneducated**: a person who did not attend any formal education.

**Improved sanitation facility:** was defined as one that hygienically separates human excreta from human contact. This includes flush or pours flush toilets flowing to a piped sewer system, septic tank, or latrine, ventilated pit latrine, pit latrine with slab, and compositing toilet. Based on the type of sanitation facility used by each household, a household was classified as having or not having an improved sanitation facility [15, 16].

**Improved water sources:** If a household used piped water (in to dwelling, compound, yard or plot, piped to neighbor, public tap/standpipe), tube well/borehole, protected well, protected spring and rain water collection for drinking purposes. If bottled water was used, the households must have had to use any of the improved water sources listed above for other purposes such as cooking and washing hands, to be considered as using improved water sources [16, 17].

**Underweight:** if an individual’s body mass index (BMI) is less than 18.5 kg/m^2^ [18].

### Data source and extraction

Initially, authorization was obtained through online request after explaining the goal of our study. Then, the data was accessed from the demography heath survey (DHS) program official database www.measuredhs.com. We used the Personal Record (PR file) data set and extracted the outcome and independent variables.

### Data processing and management

Data processing and analysis were performed using STATA 14; ArcGIS 10.1 and SaTScan 9.6 software. In order to get reliable statistical estimates, the data were weighted using sampling weight, primary sampling unit and strata before any statistical analysis. Cross tabulations and summary statistics were conducted to describe the study population.

### Spatial analysis

#### Spatial autocorrelation analysis

The term spatial autocorrelation refers to the presence of systematic spatial variation in a mapped variable. Where adjacent observations have similar data values the map shows the positive spatial autocorrelation. Where adjacent observations tend to have very contrasting values, then the map shows the negative spatial autocorrelation [19]. There are several statistical techniques for detecting its presence. In the current study, the existence of spatial autocorrelation was checked using Global Moran’s index (Moran’s I). Global Moran’s index (Moran’s I) was used to identify the presence of spatial autocorrelation. Moran’s I value ranges from-1 to 1 [20] . A value close to 1 shows a strong positive spatial autocorrelation (disease/event clustered), whereas a value close to − 1 shows a strong negative spatial autocorrelation (disease/event dispersed). If Moran’s I is close to 0, it indicates that there is no spatial autocorrelation. A statistically significant Moran’s I (*p* <  0.05) led to the rejection of the null hypothesis (anemia is randomly distributed) and showed the presence of spatial autocorrelation. Hotspot analysis was made using Gettis-OrdGi*statistics.

#### Spatial scan statistical analysis

Spatial scan statistics were applied to identify a significant Primary (most likely) and secondary cluster of anemia using Kuldorff’s SaTScan software. SaTScan™ works with a moving window and requires fixing of the window size that moves across the study area. Since the dependent variable (anaemic and not anaemic) has a Bernoulli distribution, the Bernoulli model was employed for purely spatial analysis.

To fit the Bernoulli model, Adults who were anaemic (mild, moderate, and severe) were considered as cases and those who were not anaemic were considered as controls. The default maximum spatial cluster size of < 50% of the population was considered as an upper limit, which permitted both small and large clusters to be identified and ignored clusters that contained more than the maximum limit. Areas with high Log Likelihood Ratio and significant *p*-value were taken as high anemic areas compared to areas outside of the window.

### Statistical analysis

Since the EDHS data have a hierarchical nature, adults within a cluster may be more similar to each other than with adults in another cluster. Due to this, the assumption of independence of observations and equal variance across clusters might be violated. Therefore, an advanced statistical model is required to take into account the between cluster variability to get a reliable standard error and unbiased estimate.

Furthermore, by taking the ordinal nature of the outcome variable into account, ordinal logistic regression and mixed effect ordinal logistic were fitted. Model comparison was done based on Akaike and Bayesian Information Criteria (AIC and BIC). Mixed effect model with the lowest Information Criteria (AIC and BIC) was selected**.** Adjusted Odds Ratio (AOR) with a 95% Confidence Interval (CI) and *p*-value 0.05 in the multivariable model was declared as determinant factors of anemia. The assumption of proportional odds was checked and the results tell that the assumption of proportional odds is plausible at 5% level of significance for all considered covariates in the model.

## Results

### Characteristics of study population

In this study, a total of 29,140 adults were included. Among these respondent, more than half (53.0%) of them were females, more than one-third of them were uneducated (38.51%) and poor (34.94%) in wealth index and more than three fourth (80.6%) of them were rural residents. Five thousand one hundred and forty two (20.7%) of the study participants were in the age range of 15 to 19 years. Concerning the marital status, 18,249 (62.6%) respondents were married while 8889 (30.5%) respondents were never married. Near to one-third (36.0%) of the study participants were from Oromia region. Only 6.8% of the respondents had improved toilet facility, whereas near to two-third (64.5%) of the study participants used improved drinking water (Table 2**).** Regarding, the body mass index, more than one quarter (26.4%) of the respondents were underweight.

**Table 2:** Characteristics of respondents in Ethiopia from January 18 to June 27, 2016 (*N* = 29,140)

Variables	Anemia level (weighted frequency)				Total	Percent
	Not anemic	Mild	Moderate	Severe		
**Sex**						
Female	12,674	2050	626	106	15,456	53.0
Male	11,847	1413	375	49	13,684	47.0
**Age**						
15–19	5142	699	179	20	6040	20.7
20–24	3987	508	157	27	4679	16.1
25–29	4271	563	200	27	5061	17.4
30–34	3308	501	193	37	4039	13.9
35–39	2952	429	103	18	3502	12.0
40–44	2097	362	73	12	2544	8.7
45–49	1746	233	57	14	2050	7.0
50–54	549	99	20	0	668	2.3
55–59	469	68	20	0	557	1.9
**Marital status**						
Never married	7752	892	223	22	8889	30.5
Married	15,092	2329	712	116	18,249	62.6
Widowed	424	70	19	6	520	1.8
Divorced	1253	172	46	11	1482	5.1
**Educational level**						
Uneducated	9124	1498	490	109	11,221	38.5
Primary	10,426	1487	399	36	12,348	42.4
Secondary	3264	360	68	9	3701	12.7
Higher	1664	115	44	0	1823	6.3
Don’t know	44	3	0	0	47	0.1
**Place of residence**						
Urban	5018	524	103	6	5651	19.4
Rural	19,503	2940	897	149	23,489	80.6
**Region**						
Tigray	1744	234	60	7	2045	7.0
Afar	141	44	20	2	207	0.7
Amhara	6920	779	136	11	7846	27.0
Oromia	8593	1379	432	80	10,484	36.0
Somali	404	150	112	22	688	2.3
Benishangul	243	28	7	0	278	1.0
SNNPR	5051	695	193	28	5967	20.4
Gambella	66	10	2	0	78	0.3
Harari	43	7	3	1	54	0.2
Addis Ababa	1205	116	28	1	1350	4.6
Dire Dawa	113	21	8	1	143	0.5
**Wealth index**						
Poor	8115	1445	522	101	10,183	35.0
Middle	4863	689	190	12	5754	19.7
Rich	11,543	1330	288	42	13,203	45.3
**Source of drinking water**						
Improved	16,142	2071	528	54	18,795	64.5
Not improved	8379	1392	473	101	10,345	35.5
**Type of toilet facility**						
Improved	1710	209	50	3	1972	6.8
Not improved	22,811	3254	951	152	27,168	93.2
**BMI**						
Underweight	6390	963	300	52	7705	26.4
Normal	16,715	2324	655	90	19,784	67.9
Overweight	1124	135	40	13	1313	4.5
Obese	292	41	5	1	339	1.2

*BMI* Body Mass Index, *SNNPR* Southern Nation and Nationality and Peoples Regions

### Spatial analysis of anemia

#### Spatial distribution of anemia

**Fig. 1:**
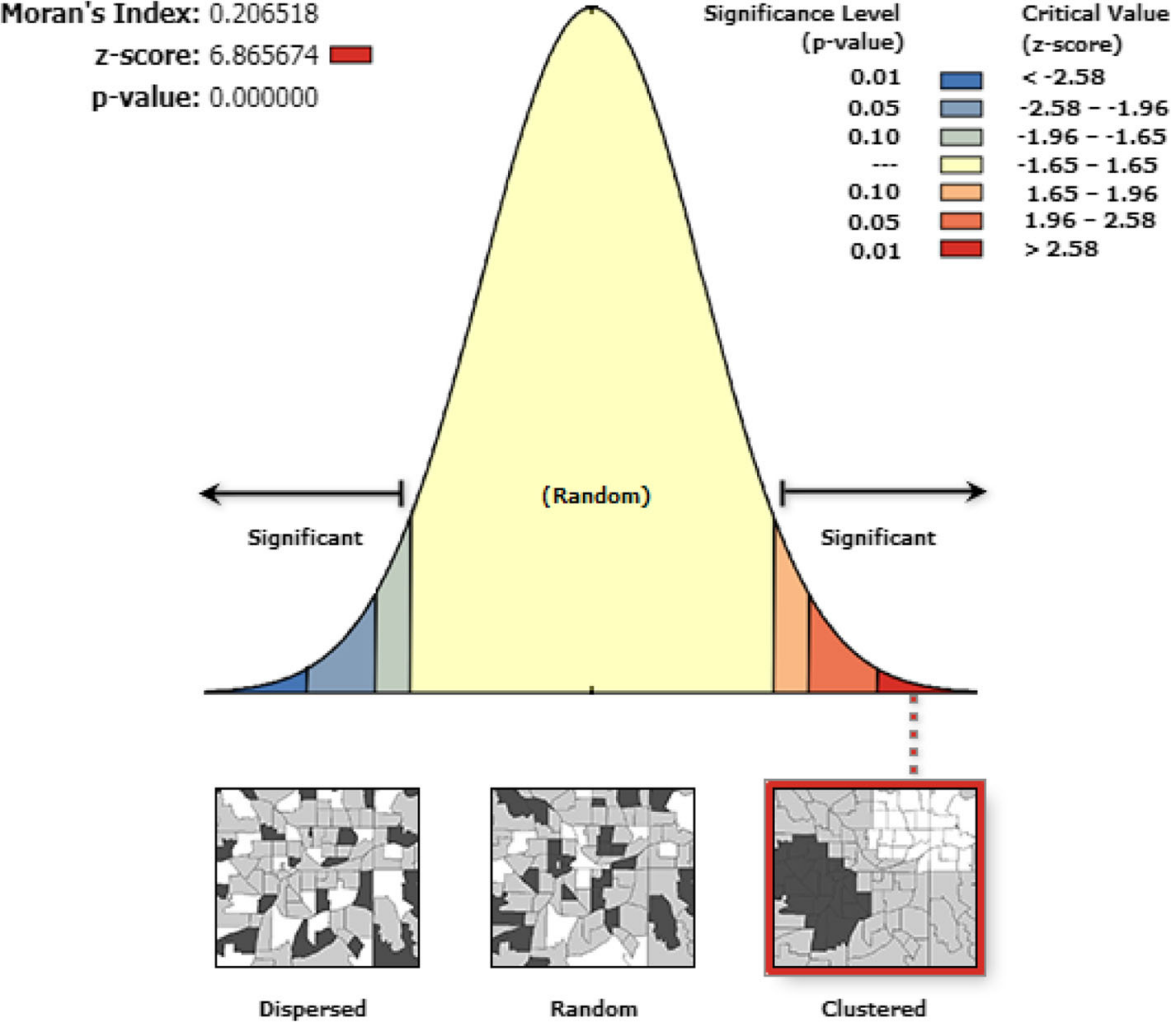
Spatial autocorrelation analysis of anemia among adults age 15–59 in Ethiopia, 2016

**Fig. 2:**
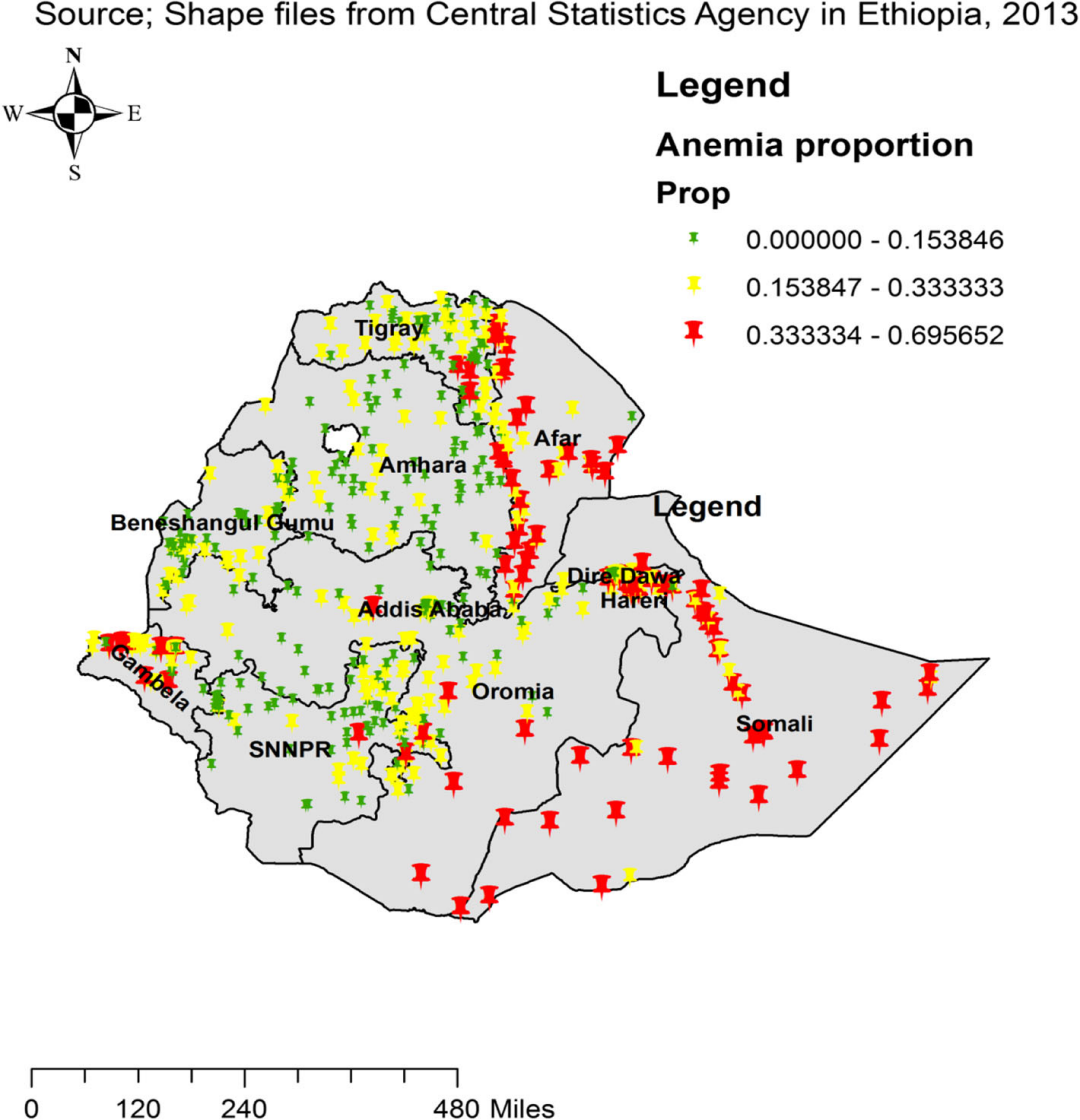
Spatial distribution of anemia across regions among adults age 15–59 in Ethiopia, 2016

The spatial distribution of anemia in Ethiopia among adults age 15–59 was identified to be clustered (Global Moran’s I = 0.81, *p* value <  0.0001). The outputs of were automatically produced keys on the right and left sides of each panel. Given the z-score of 6.9, it is clear that there is less than 1% likelihood that this clustered pattern could be the result of chance. The bright red and blue colours to the end tails indicate an increased significance level (Fig. 1). The highest proportion of anemia were found in the Somalia, Afar and Gambella regions, whereas low proportion of anemia were located in the Amhara, Tigray, and northern part of SNNPR, central, West and East Oromia and Benishangul regions **(**Fig. 2**)**.

### Gettis-OrdGi statistical analysis of anemia

**Fig. 3:**
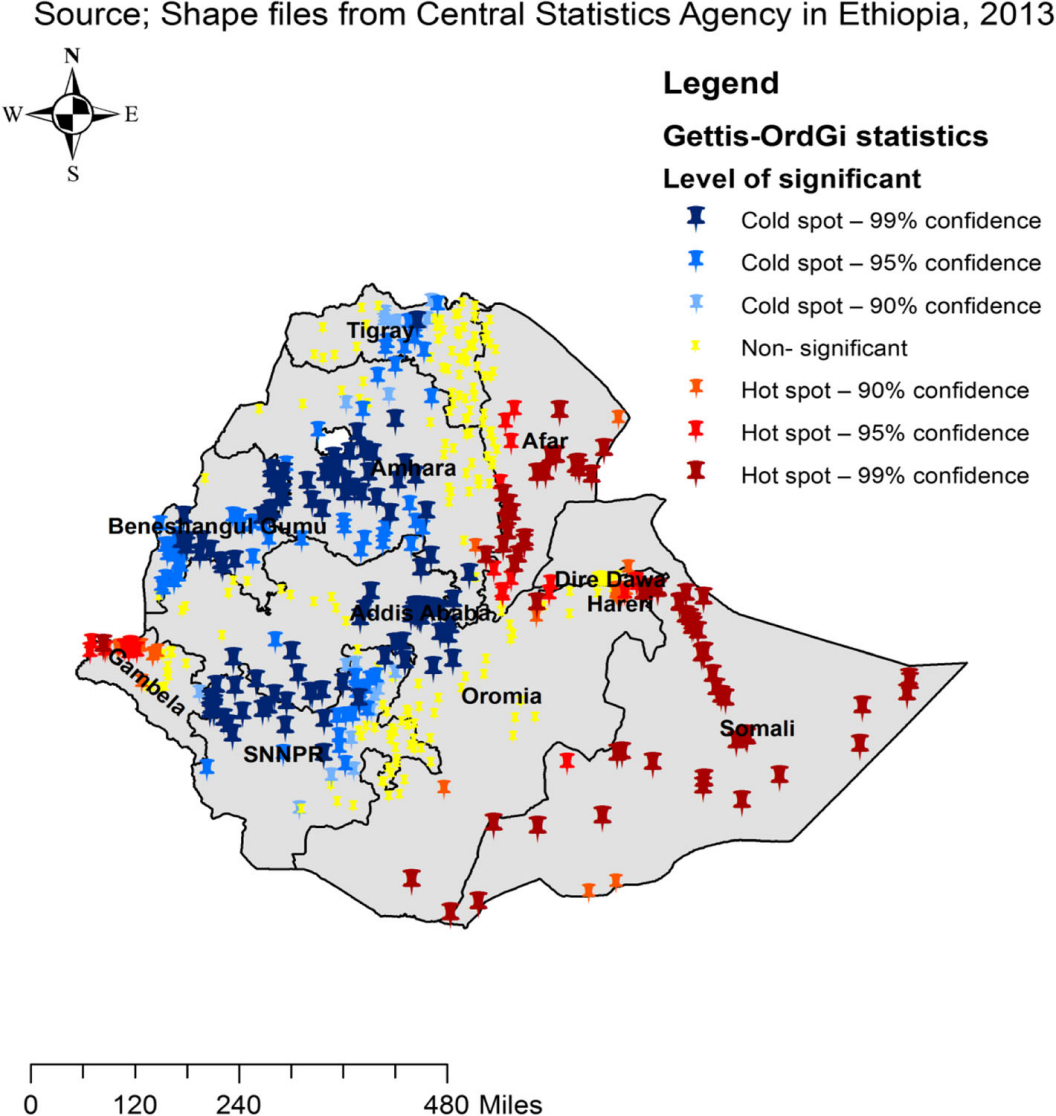
Hotspot and Cold areas anemia across regions among adults age 15–59 in Ethiopia, 2016

Based on the Gettis-OrdGi statistical analysis, this study identified hotspots and cold spot areas of anemia in Ethiopia among adults aged 15–59. Accordingly, the red colors indicate the significant hotspot area (higher cluster of anemia), which were found in the Somalia, Afar, Gambella, Dire Dewa and Harari regions. In contrast, the blue color indicates significant cold spot areas (low cluster of anemia), located in the Amhara, central Tigray and northern part of SNNPR, central and west east Oromia and Benishangul regions **(**Fig. 3**).**

### Spatial SaTScan analysis of Anemia (Bernoulli based model)

**Fig. 4:**
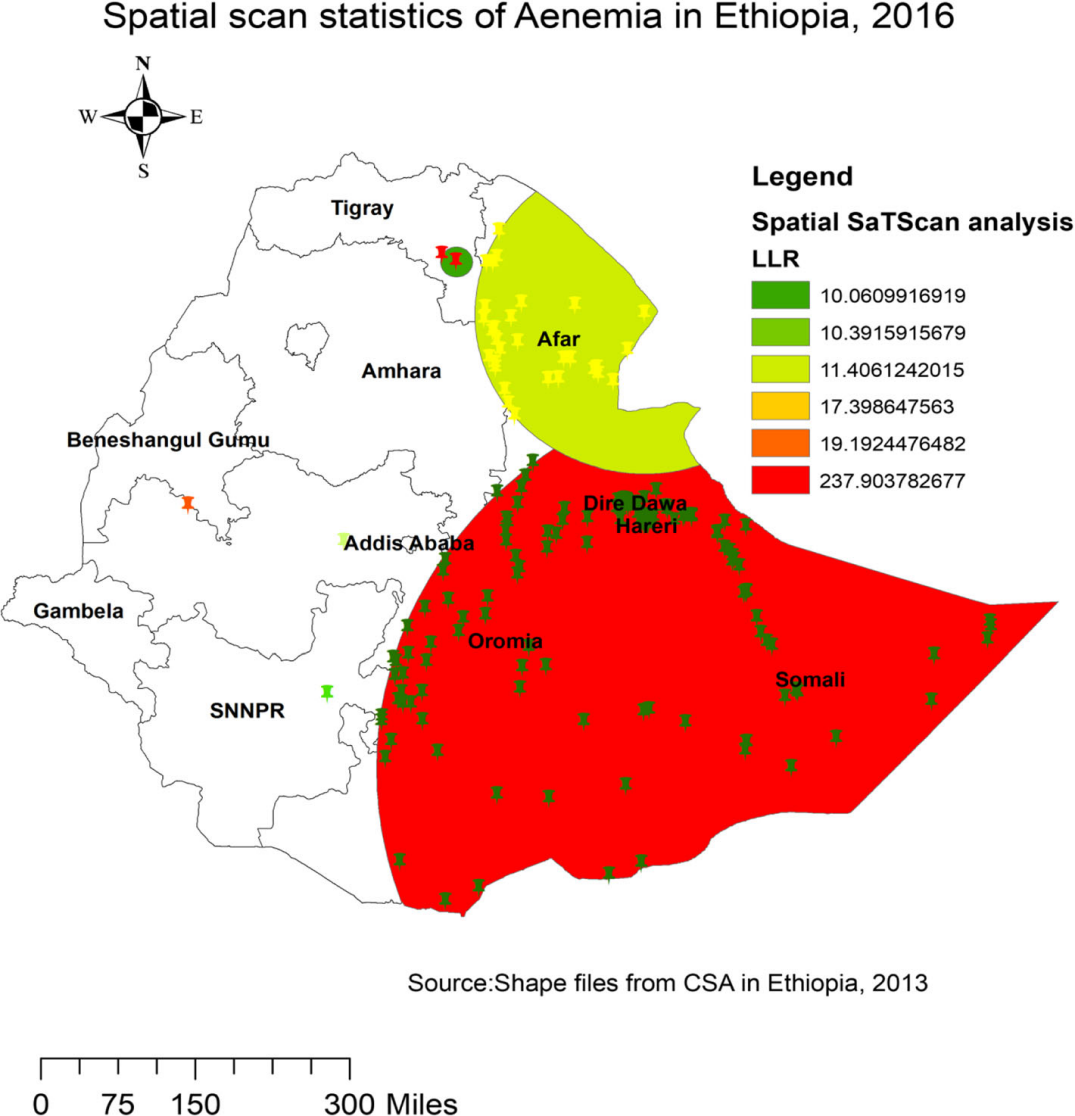
Primary and secondary clusters of Anemia among adults age 15–59 in Ethiopia, 2016

A spatial scan statistical analysis recognized a total of 193 significant primary and secondary clusters. Among these, 192 clusters were primary (most likely) clusters which were located in the Somalia, Dire Dewa, Harari and eastern part of Oromia region at 5.589269 N and 44.175032 E, with 647.61 km radius, a Relative Risk (RR) of 1.89, and Log-Likelihood Ratio (LRR) of 237.90, at *p*-value*<* 0.01 **(**Table 3**)**. This tells us that adults within the spatial window had 1.89 times higher risk of experiencing anemia as compared to adults outside the spatial window. The secondary clusters were found in border areas between SNNPRs and Oromia regions (Fig. 4).

**Table 3:** Significant spatial clusters with high rate of Anemia among adults age 15–59 in Ethiopia, 2016

Cluster	Enumeration area (cluster) identified	Coordinate (radius)	Population	Case	RR	LLR	***P***-value
1	138, 164, 85, 358, 146, 492, 92, 490, 543, 278, 171, 198, 95, 318, 77, 187, 497, 556, 520, 629, 521, 588, 553, 458, 480, 208, 214, 251, 573, 239, 269, 116, 22, 394, 378, 630, 568, 33, 277, 286, 527, 289, 64, 439, 57, 186, 8, 210, 472, 452, 377, 454, 513, 436, 501, 212, 68, 580, 622, 483, 566, 133, 587, 194, 240, 500, 321, 418, 58, 115, 29, 44, 534, 179, 257, 387, 157, 397, 56, 607, 228, 28, 614, 396, 60, 393, 357, 419, 443, 173, 238, 329, 1, 288, 383, 495, 381, 610, 473, 372, 453, 242, 523, 281, 642, 166, 311, 307, 30, 557, 202, 441, 594, 613, 352, 74, 519, 380, 535, 273, 471, 631, 151, 5, 185, 444, 111, 514, 282, 27, 390, 606, 493, 385, 224, 467, 644, 43, 363, 190, 546, 101, 140, 25, 93, 7, 476, 412, 529, 245, 123, 333, 506, 319, 422, 122, 491, 562, 213, 34, 71, 82, 518, 49, 26, 619, 51, 405, 524, 230, 564, 468, 576, 313, 365, 316, 589, 39, 438, 601, 149, 398, 336, 12, 125, 391, 522, 600, 445, 578, 484, 135	(5.589269 N, 44.175032 E) / 647.61 km	6265	1638	1.89	237.90	< 0.001
2	180	(6.720108 N, 37.624880 E) / 0 km	113	45	2.56	19.19	< 0.001

*LLR* Likelihood ratio, *RR* Relative risk

### Determinant factors of anemia

Based on multivariable mixed-effects ordinal logistic regression model, sex, marital status, educational level, place of residence, region, wealth index and body mass index were significantly associated with the level of anemia among adults in Ethiopia at *p*-value 0.05 (Table 4).

**Table 4:** Bi-variable and multivariable mixed-effects ordinal logistic regression model of anemia among adults age 15–59 in Ethiopia, 2016. ((*N* = 28,450))

Variables	Crude odds ratio		Adjusted odds ratio		
	OR	95% CI	OR	95% CI	*P*-value
**Sex**					
Male	Ref	Ref	Ref	Ref	Ref
Female	1.57	[1.47, 1.68]	1.53	**[1.42, 1.66]**	**< 0.001**
**Age**					
15–19	Ref	Ref	Ref	Ref	Ref
20–24	1.01	[0.90, 1.12]	0.96	[0.85, 1.08]	0.49
25–29	1.12	[1.01, 1.25]	1.04	[0.91, 1.18]	0.59
30–34	1.28	[1.14, 1.43]	1.12	[0.97, 1.29]	0.12
35–39	1.15	[1.02, 1.29]	1.01	[0.86, 1.17]	0.95
40–44	1.16	[1.02, 1.32]	1.03	[0.88, 1.21]	0.70
45–49	1.02	[0.88, 1.18]	0.91	[0.76, 1.08]	0.29
50–54	0.99	[0.78, 1.24]	1.13	[0.87, 1.46]	0.37
55–59	1.06	[0.82, 1.37]	1.18	[0.89, 1.57]	0.26
**Marital status**					
Married	Ref	Ref	Ref	Ref	Ref
Never married	0.74	[0.69, 0.80]	0.86	**[0.77, 0.96]**	**0.01**
Widowed	1.13	[0.91, 1.40]	0.99	[0.80, 1.24]	0.98
Divorced	1.02	[0.88, 1.17]	0.96	[0.83, 1.11]	0.62
**Educational level**					
Uneducated	Ref	Ref	Ref	Ref	Ref
Primary	0.72	[0.67, 0.78]	0.90	**[0.83, 0.98]**	**0.02**
Secondary	0.58	[0.51, 0.65]	0.81	**[0.71, 0.92]**	**0.02**
Higher	0.47	[0.40, 0.55]	0.71	**[0.60, 0.84]**	**< 0.001**
Don’t know	0.33	[0.12, 0.95]	0.46	[0.16, 1.32]	0.15
**Place of residence**					
Urban	Ref	Ref	Ref	Ref	Ref
Rural	1.93	[1.64, 0.27]	1.53	**[1.23, 1.81]**	**< 0.001**
**Region**					
Oromia	Ref	Ref	Ref	Ref	Ref
Tigray	0.79	[0.62, 1.02]	0.80	[0.64, 1.02]	0.06
Afar	2.38	[1.84, 3.09]	2.04	**[1.61, 2.59]**	**< 0.001**
Amhara	0.61	[0.47, 0.77]	0.60	**[0.48, 0.76]**	**< 0.001**
Somali	3.76	[2.95, 4.79]	3.43	**[2.75, 4.28]**	**< 0.001**
Benishangul	0.66	[0.50, 0.87]	0.63	**[0.49, 0.81]**	**< 0.001**
SNNPR	0.75	[0.59, 0.96]	0.76	**[0.60, 0.94]**	**0.01**
Gambella	1.02	[0.78, 1.34]	1.10	[0.86, 1.41]	0.44
Harari	1.13	[0.85, 1.51]	1.48	**[1.13, 1.93]**	**0.01**
Addis Ababa	0.53	[0.40, 0.69]	1.01	[0.77, 1.34]	0.92
Dire Dawa	1.18	[0.89, 1.56]	1.68	**[1.28, 2.19]**	**< 0.001**
**Wealth index**					
Poor	Ref	Ref	Ref	Ref	Ref
Middle	0.811	[0.73, 0.91]	0.92	[0.83, 1.03]	0.17
Rich	0.59	[0.53, 0.66]	0.77	[**0.69, 0.86]**	**< 0.001**
**Source of drinking water**					
Not improved	Ref	Ref	Ref	Ref	Ref
Improved	0.87	[0.80, 0.96]	1.05	[0.96, 1.14]	0.32
**Type of toilet facility**					
Not improved	Ref	Ref	Ref	Ref	Ref
Improved	0.94	[0.84, 1.07]	1.01	[0.90, 1.15]	0.81
**BMI**					
Normal	Ref	Ref	Ref	Ref	Ref
Underweight	1.09	[1.01, 1.17]	1.15	**[1.06, 1.24]**	**< 0.001**
Overweight	0.94	[0.81, 1.09]	0.88	[0.76, 1.02]	0.09
Obese	0.99	[0.77, 1.27]	0.88	[0.68, 1.14]	0.33
**Random intercept**					
*Var (cons)*	–	–	0.28	[0.23, 0.34]	–

*AOR* Adjusted Odd ratio, *COR* Crude Odd Ratio, *CI* Confidence interval, *SNNPR* Southern Nation and Nationality and Peoples Regions

The odds of having severe anemia among female adults (compared to moderate, mild or non-anemic) were 1.53 times higher than male adults while keeping other variables constant (OR = 1.53; 95% CI: 1.42, 1.66). Making other variables constant, the likelihood of experiencing severe anemia among adults that were never married (relative to moderate, mild or non-anemic) were 0.86 times lower than married adults (OR = 0.86; 95% CI: 0.77, 0.96).

Educational level was an important variable that showed significant association with level of anemia among adults in Ethiopia. Holding other variables constant, the chances of uneducated adults developing severe anemia (versus moderate, mild or non-anemic) were 0.90, 0.81, 0.71 times lower than primary, secondary and higher educated adults respectively.

The odds of having severe anemia among rural adult (against moderate, mild or non-anemic) were 1.53 times higher than urban adults while adjusting for other variables (OR = 1.53; 95% CI: 1.23, 1.81). Keeping other variables constant, the likelihoods of underweight adult having severe anemia (opposed to moderate, mild or non-anemic) were 1.15 times higher than normal adults (OR = 1.15; 95% CI: 1.06, 1.24).

The likelihoods of rich adult developing severe anemia (compared to moderate, mild or non-anemic) were 0.77 times lower than poor adults (OR = 0.77; 95% CI: 0.69, 0.86) while holding other variables constant and random effect at level one.

Regarding region, Adults residing in Afar, Somali, Harari and Dire Dawa were 1.68, 3.43, 1.48 and 2.04 times more likely to be severe anaemic (relative to moderate, mild or non-anemic) than adults residing in Oromia respectively whereas the odds of severe anaemic (opposed to moderate, mild or non-anemic) were decreased by 40, 37 and 24% among adults residing in Amhara, Benishangul and SNNPR as compared with adult residing in Oromia respectively.

## Discussion

This study is aimed to investigate the spatial distribution and determinants of anemia among adults in Ethiopia. The spatial analysis result showed that the spatial distribution of anemia among adults was significantly varied across the country. In multivariable mixed-effect ordinal regression analysis; sex, marital status, educational level, place of residence, region, wealth index and body mass index were significant predictors of the level of anemia among adults in Ethiopia.

The present study documented that the spatial distribution of anemia among adults significantly varied across the country; significant hotspot areas of anemia were identified in the Somalia, Afar, Gambella, Dire Dewa and Harari regions. This spatial variation might be due to the difference in socioeconomic status, infectious disease risk (such as malaria, hookworm and etc.), dietary diversity and food security [21].

According to the current study, the odds of female adults having severe anemia (compared to moderate, mild or non-anemic) were higher than male adults. This finding is in line with studies conducted in India [22] and United States of America [23]. Such similarity of finding might be due to the fact that women experienced more blood loss through menstruation and had greater demand blood supply for the developing fetus during pregnancy.

In agreement with another study [24], the current study revealed that the chances of uneducated adults developing severe anemia (versus moderate, mild or non-anemic) were higher than the chances of educated adults. The possible explanations for such results might be due to low socio-economic status, risky lifestyle and low diseases prevention knowledge and skills among uneducated adults as compared to educated adults.

The present study documented that the odds of rural adults experiencing severe anemia (against moderate, mild or non-anemic) were higher than odds of urban adults and this is of course congruent with other reports [24, 25]. The possible reasons might be rural adults are more likely to have low socioeconomic status, low chance of accessing iron-rich foods and lack of adequate nutrition information as compared to urban adults [24].

In line with another study [25], the present study reported that likelihoods of rich adult developing severe anemia (compared to moderate, mild or non-anemic) were lower than likelihoods of poor adults. Such result indicates that poor adults have less access to nutritious food and fall sick more frequently as compared to the rich adults. This finding may not be surprising because maintaining food security is a big issue among poor families [26].

According to the current study, the likelihoods of underweight adults having severe anemia (opposed to moderate, mild or non-anemic) were higher than normal adults. Even though a higher body mass index may not always show proper micronutrient consumption, an underweight (BMI < 18.5 g/m^2^) person is more likely to have the other concomitant comorbidity illness and scarce some essential micronutrients that may be related with anemia [25].

The current study has some strengths and limitations that need to be kept in mind while interpreting the result. The first strength of the current study was using large population-based data with a large sample size, which is representative at national and regional levels, so it can be generalized to adults in Ethiopia aged15 to 59. Secondly, the combined use of both ArcGIS and Sat Scan statistical tests facilitated to identify similar and statistically significant areas with a high cluster of anemia (hot spot area). Furthermore, by considering the ordinal nature of the outcome variable and the cluster nature of data, the current study applied an advanced model (mixed effect ordinal logistic regression) to get reliable standard errors and parameter estimates.

The first limitation of the present study was that the location of data values was displaced up to 2 km for urban and up to 5 km for rural areas to ensure respondent confidentiality; thus, this was the challenge to know the exact cases of’ location. Since the current study used secondary data, some important variable like dietary intake, *Plasmodium falciparum* parasite rate and hookworm were not included in the analysis.

## Conclusion

A significant clustering of anemia among adults aged 15–59 was found in Ethiopia and the significant hotspot areas with high cluster anemia were identified in Somalia, Afar, Gambella, Dire Dewa and Harari regions. Besides, sex, marital status, educational level, place of residence, region, wealth index and BMI were significant predictors of anemia. Thus, active public health measurement along with nutritional education should be designed in the identified hotspot areas and for risk groups in order to decrease the incidence of anemia.

## Data Availability

The datasets supporting the conclusions of this article are available upon request to the corresponding author.

## Abbreviations

AIC: Akaike Information Criteria
AOR: Adjusted Odds Ratio
BIC: Bayesian Information Criteria
BMI: Body mass index
CI: Confidence
CSA: Central Statistics Agency
DHS: Demography heath survey
EAs: Enumeration Areas
EDHS: Ethiopian Demographic and Health Survey
SNNPR: Southern Nation and Nationality and Peoples Regions
